# Requirement of HIV-1 Vif C-terminus for Vif-CBF-β interaction and assembly of CUL5-containing E3 ligase

**DOI:** 10.1186/s12866-014-0290-7

**Published:** 2014-11-26

**Authors:** Hong Wang, Guoyue Lv, Xiaohong Zhou, Zhaolong Li, Xin Liu, Xiao-Fang Yu, Wenyan Zhang

**Affiliations:** Institute of Virology and AIDS Research, First Hospital of Jilin University, No 519. East Minzhu Avenue, Changchun, Jilin Province China; Department of Hepatobiliary and Pancreatic Surgery, First Hospital of Jilin University, No 519. East Minzhu Avenue, Changchun, Jilin Province China; Department of Molecular Microbiology and Immunology, Johns Hopkins Bloomberg School of Public Health, 615 N. Wolfe Street, Baltimore, MD USA

**Keywords:** HIV-1 Vif, CBF-β, C-terminus, APOBEC3

## Abstract

**Background:**

Human immunodeficiency virus type 1 (HIV-1) Vif hijacks an E3 ligase to suppress natural APOBEC3 restriction factors, and core binding factor β (CBF-β) is required for this process. Although an extensive region of Vif spanning most of its N-terminus is known to be critical for binding with CBF-β, involvement of the Vif C-terminus in the interaction with CBF-β has not been fully investigated.

**Results:**

Here, through immunoprecipitation analysis of Vif C-terminal truncated mutants of various lengths, we identified that CBF-β binding requires not only certain amino acids (G126A, E134A, Y135A and G138A) in the HCCH region but also the HCCH motif itself, which also affects the Vif-mediated suppression of APOBEC3G/APOBEC3F (A3G/A3F). These mutants still maintained interactions with substrate A3G or A3F as well as other cellular factors ElonginB/C (ELOB/C), indicating that their structures were not functionally affected. Moreover, by determining that the BC box also is necessary for CBF-β interaction *in vivo*, we speculate that binding to ELOB/C induces conformational changes in Vif, facilitating its interaction with CBF-β and consequent interaction with CUL5.

**Conclusions:**

These results provide important information on the assembly of the Vif-CUL5-E3 ubiquitin ligase. Identification of the new binding interface with CBF-β at the C-terminus of HIV-1 Vif also provides novel targets for the development of HIV-1 inhibitors.

## Background

Polynucleotide cytidine deaminases comprise a large family of proteins, including AID, APOBEC1, APOBEC2, APOBEC4 and seven APOBEC3 proteins (A3A, A3B, A3C, A3DE, A3F, A3G, A3H). APOBEC3 proteins were discovered to have antiviral or anti-retrotransposon activities of varying degrees [1-13]. To counteract these host restriction factors, the HIV-1 Vif protein hijacks host Cullin5 (CUL5), ElonginB/ElonginC (ELOB/C) and a newly identified host factor CBF-β to form an E3 ubiquitin ligase to induce APOBEC3 protein ubiquitination and degradation [14-26].

**Figure 1:**
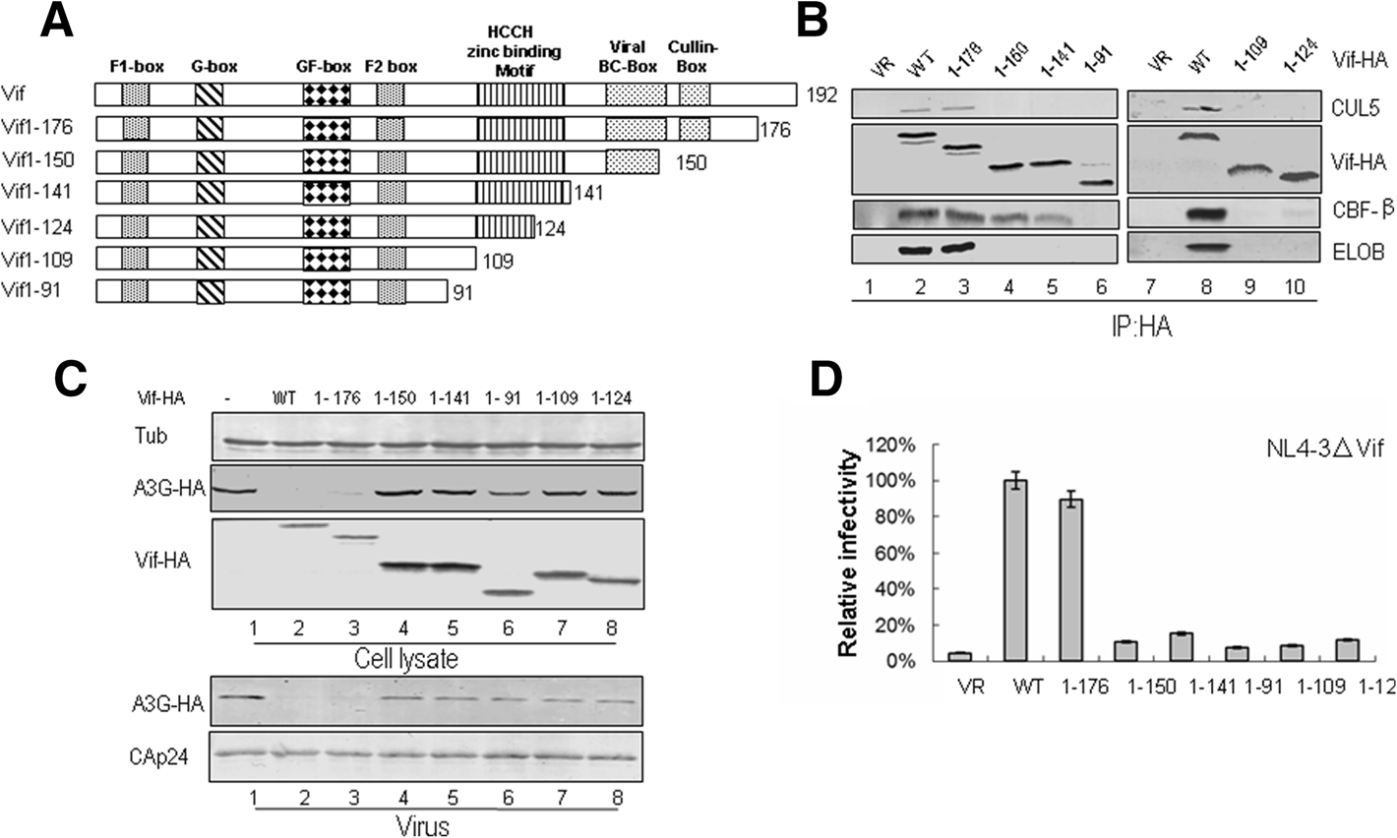
**Effects of C-terminal truncations on HIV-1 Vif function. (A)** Construction of C-terminal truncated Vif mutants with an N-terminal HA tag. **(B)** Interactions of various truncated Vif mutants with cellular factors. HEK293T cells were transfected with VR1012 as a control vector or WT or truncated Vif mutants as indicated. Cells were harvested 48 h later and subjected to immunoprecipitation analysis using the anti-HA antibody conjugated to agarose beads. Co-precipitated proteins were analyzed by Western blotting against Vif-HA, CUL5, CBF-β and ELOB. **(C)** Effects of WT and truncated Vif proteins on A3G degradation and virion packaging. HEK293T cells were co-transfected with NL4-3ΔVif and A3G along with VR1012 as a control vector or WT or various truncated Vifs as indicated. A3G expression was assessed by Western blotting against A3G-HA, Vif-HA and tubulin as loading control. A3G packaging was evaluated by Western blotting against A3G-HA and CAp24 after virus was purified from the supernatant of cell cultures. **(D)** Effects of WT and truncated Vif proteins on antiviral activity of A3G. HIV-1 viruses were produced as described for panel C. Virus infectivity was assessed using MAGI indicator cells, with the virus infectivity in the presence of WT Vif set to 100%. Error bars represent the standard deviation from triplicate wells.

HIV-1 Vif is a 23-kDa protein with 192 residues. Previous studies have identified multiple functional domains in HIV-1 Vif (Figure 1A) [27,28]. In its C-terminus, a virus-specific region, termed BC box, is required for interaction with ELOB/C [29-32]. Furthermore, a highly conserved H-X_5_-C-X_17-18_C_3-5_-H motif (HCCH motif) upstream of the BC box, the CUL5 box downstream of the BC box and an N-terminal motif are responsible for CUL5 binding [32-40]. The N-terminal domain of Vif is important for binding to its APOBEC3 substrate [41-47]. The Vif Y^40^RHHY^44^ motif is specific for A3G binding (G box) [46], while D^14^RMR^17^ and T^74^GERxW^79^ both are specific for A3F binding (F box) [41,46]. The common binding sites for both A3G and A3F are W^21^KSLVK^26^, V^55^xIPLx_4-5_LxΦYWxL^72^ and Y^69^xxL^72^ (GF box) [41-43,45].

As a newly identified Vif regulator in 2011, CBF-β has been shown to be critical for Vif-mediated degradation of APOBEC3 family proteins [20-24,48,49]. CBF-β increases the stability of HIV-1 Vif [20,23,24] and specifically interacts with this viral protein to control its binding to CUL5 [20,22,23]. CBF-β also increases Vif solubility when co-expressed *in vitro* [23].

Recent studies suggested that the N-terminal amino acids of Vif, including dispersed and conserved hydrophobic amino acids, are important for binding with CBF-β [50-52]. However, whether the C-terminus of HIV-1 Vif is also required for its interaction with CBF-β is unclear. In the current study, we first mapped the critical region for CBF-β binding at the C-terminus of HIV-1 Vif by using the NL4-3 strain Vif sequence (Vif) to create C-terminal truncated mutants of various lengths and found that the Vif 1-141 truncated mutant, but not Vif 1-124, Vif 1-109 or Vif 1-91 truncated mutant, still maintained a certain degree of binding to CBF-β. These results showed that certain amino acids between positions 141 and 124 are required for the CBF-β interaction. Subsequently, we screened a series of single-site Vif mutants in this region. Our results suggested that the mutations Y135A, G138A, G126A and E134A in this region affect the suppressive function of Vif on A3G/A3F antiviral activity, which is due to loss of the ability to interact with CBF-β. Moreover, the data also indicated that the HCCH motif itself affects the binding with CBF-β. Thus, we have identified several amino acids at the C-terminus of HIV-1 Vif that are important for the interaction with CBF-β and Vif function, which may be novel targets for the development of HIV-1 inhibitors.

## Methods

### Plasmid construction

The Vif mutant infectious molecular clone (pNL4-3∆Vif) was obtained from the AIDS Research Reagents Program, Division of AIDS, National Institute of Allergy and Infectious Diseases, National Institutes of Health (NIH-ARRRP). The VR1012 vector was generously provided by Vical. Vif-HA was constructed by PCR amplifying codon optimized Vif from NL4-3 (residues 1-192) with a C-terminal HA tag and cloning the product into VR1012 via *Eco*RI and *Bam*HI sites. The following primers were used to create Vif-HA: forward 5′-CTCTCTGAATTCATGGAGAACCGGTGG-3′; reverse 5′-ATGGATCCCTACGCGTAATCTGGGACGTCGTAAGGGTAGTGTCCATTCATTG-3′ (HA). All truncated Vif mutants (1-176, 1-150, 1-141, 1-91, 1-109, 1-124) were amplified from the WT Vif-HA plasmid and inserted into VR1012 via *Eco*RI and *Bam*HI sites. Plasmids for HIV-1 Vif mutants H108A, C114A, L125A, G126A, R127A, I128A, V129A, S130A, P131A, R132A, C133A, E134A, Y135A, Q136A, A137S, G138A, H139A, N140A and K141A also were constructed from the WT Vif plasmid by site-directed mutagenesis. Plasmids for SIVmac Vif mutants C116S, C135S and C116/135S were derived from the SIVmac Vif-HA plasmid by site-directed mutagenesis. The V5-tagged human A3F-expressing vector pcDNA3-A3F-V5 was a kind gift from Yonghui Zheng and B. Matija Peterlin at the University of California, San Francisco [3]. The expression vectors pc-hA3G-HA, pc-hA3G-V5, SIVtan Vif-HA, SIVtan Vif SLQ-AAA, H111L, C117S, C135S, H141L and SIVmac Vif-HA were described previously [14,22].

### Cell culture, transfection and antibodies

The human HEK293T (ATCC, catalog no. CRL-11268) and MAGI (NIH-ARRRP, catalog no. 3522) cells from Julie Overbaugh [53] were maintained in Dulbecco’s modified Eagle’s medium (DMEM, Invitrogen) with 10% fetal bovine serum and penicillin/streptomycin (D-10 medium) and passaged upon confluence. DNA transfection with PEI reagent was carried out according to the manufacturer’s instructions (Polyscience, catalog no. 23966-2). The following antibodies or antisera were used in this study: anti-CUL5 (H-300) rabbit polyclonal antibody (Santa Cruz Biotechnology, sc-13014), anti-Vif rabbit polyclonal antibody (NIH-ARRRP, catalog no 2221), CAp24 monoclonal antibody (NIH-ARRRP, catalog no. 1513), anti-HA rabbit polyclonal antibody (Santa Cruz Biotechnology, 71-5500), anti-CBF-β monoclonal antibody (Santa Cruz Biotechnology, sc-166142), anti-ElonginB (FL-118) rabbit polyclonal antibody (Santa Cruz Biotechnology, sc-1144), anti-V5 monoclonal antibody (Invitrogen, R960-25), anti-β-tubulin monoclonal antibody (Tianjin Sanjian, catalog no. DKM9003). MG132 was purchased from Sigma (catalog no. C2211).

### Virus purification, viral infectivity (MAGI) assay and A3G/A3F degradation

For APOBEC3 (A3G or A3F) degradation and APOBEC3 proteins packaging assays, HEK293T cells were transfected with 0.5 μg of NL4-3ΔVif, 0.1 μg of negative control vector VR1012 or WT or mutant Vif and 0.5 μg of A3G-HA or A3F-HA in six-well plates. Viruses were produced by transfecting HEK293T cells and harvesting the supernatant, while the cells were reserved for detection of A3G or A3F degradation. Viruses in cell culture supernatants were cleared of cellular debris by centrifugation and filtration through a 0.22-μm-pore size membrane (Millipore). Virus particles were then concentrated by ultracentrifugation. Viral pellets were resuspended in lysis buffer (PBS containing 1% Triton X-100 and Complete protease inhibitor cocktail [Roche]). Viral lysates were analyzed by Western blotting.

Viral infectivity was determined by the MAGI assay as follows [53]. MAGI cells were prepared in 24-well plates in D-10 medium 1 day before infection. Cells at 30–40% confluency on the day of infection were infected by removing the medium from each well and adding dilutions of virus in a total volume of 500 μl of complete DMEM with 20 μg of DEAE-dextran per well. After 2 h of incubation at 37°C in a 5% CO_2_ incubator, 500 μl of complete DMEM was added to each well, and the cells were incubated for 48 h under the same conditions. The supernatants were removed, and 500 μl of fixing solution (1% formaldehyde, 0.2% glutaraldehyde in PBS) was added. After 5 min of incubation, the cells were washed twice with PBS. The staining solution (20 μl of 0.2 M potassium ferrocyanide, 20 μl of 0.2 M potassium ferricyanide, 2 μl of 1 M MgCl_2_ and 10 μl of 40 mg/ml 5-bromo-4-chloro-3-indolyl-β-D-galactopyranoside [X-Gal]) was added. Cells were incubated for 2 h at 37°C in a non-CO_2_ incubator. Staining was stopped by removing the staining solution, and the cells were thoroughly washed twice with PBS. β-galatosidase activity is under the control of the HIV-1 LTR promoter, which is trans-activated in this system. Positive blue dots, which indicate the presence of integrated virus, were counted, and viral infectivity was determined after normalizing the amount of input virus to the p24 antigen content. All results represent infections performed in triplicate.

### Immunoprecipitation and Western blot analysis

To assess the binding of Vif mutants to CBF-β and other factors in the CUL5 E3 ligase complex, HEK293T cells were transfected with 1 μg of WT or mutant Vif expression vectors in six-well plates. To test the interaction between Vif and A3G or A3F, the ratio of Vif to A3G or A3F was 1:3 (0.5 μg and 1.5 μg), and MG132 was added to the cells at the final concentration of 10 μM 12 h before harvest. Harvested cells were re-suspended in lysis buffer [50 mM Tris-HCL (pH 7.5),150 mM NaCl, 0.5% (v/v) NP-40 and Complete protease inhibitor cocktail tablets] and incubated at 4°C for 30 min, followed by centrifugation at 13,000 rpm for 30 min. For HA-tag immunoprecipitation, pre-cleared cell lysates were mixed with anti-HA antibody-conjugated agarose beads (Roche) and incubated at 4°C for 3 h. Samples were then washed six times with washing buffer [20 mM Tris-HCl (pH 7.5), 100 mM NaCl, 0.1 mM EDTA, 0.05% (v/v) Tween-20]. Proteins binding to the beads were eluted with elution buffer (0.1 mM glycine-HCl, pH 2.0) or 4× loading buffer. The eluted materials were subsequently analyzed by Western blot.

## Results

### A novel region in HCCH motif of HIV-1 Vif is required for maintaining Vif-CBF-β interaction

Some N-terminal amino acids in HIV-1 Vif have been shown to be important for the interaction with CBF-β [22,24,50-52]. In order to determine whether the C-terminus of Vif is critical for CBF-β binding, we constructed a series of expression vectors containing various C-terminal truncated NL4-3 Vif sequences with a HA tag at the C-terminus (Figure 1A). We transfected HEK293T cells with the negative control vector VR1012, wild-type (WT) Vif or various truncated Vif mutants as indicated in Figure 1B. At 48 h after transfection, cells were harvested, lysed and then loaded onto HA agarose-conjugated beads for immunoprecipitation. The WT and truncated Vif 1-176 mutant proteins were efficiently co-immunoprecipitated with ELOB/C, CBF-β and CUL5 (Figure 1B, lanes 2, 3 and 8). These factors were not co-immunoprecipitated in the absence of Vif, indicating the specificity of the assay (Figure 1B, lanes 1 and 7). Vif 1-150 and Vif 1-141 all lost the ability to interact with ELOB/C and CUL5, but they could still bind to CBF-β with a subtle decrease in reactivity compared to the WT Vif (Figure 1B, lanes 4 and 5). However, Vif 1-91, Vif 1-109 and Vif 1-124 lost the ability to interact with ELOB/C, CUL5 and CBF-β (Figure 1B, lanes 6, 9 and 10). Thus, Vif truncated mutants that retained the whole HCCH domain (i.e., Vif 1-176, Vif 1-150 and Vif 1-141) kept some degree of interaction with CBF-β, but those that had an impaired HCCH domain (i.e., Vif 1-124, Vif 1-109, Vif 1-91) all lost the interaction with CBF-β.

Taken together, these data showed that Vif truncated mutants lacking the BC box and containing the HCCH motif still maintained the ability to interact with CBF-β, but those that had a disrupted HCCH region lost the ability to interact with CBF-β. Therefore, we speculated that certain amino acids in the HCCH region are required for the Vif-CBF-β interaction.

We further examined the effects of WT and truncated Vif mutants on A3G degradation, A3G incorporation into virions and viral infectivity. HEK293T cells were co-transfected with the Vif mutant infectious molecular clone NL4-3ΔVif and A3G expression vector along with VR1012 as a negative control vector or expression vector for WT or different truncated Vif proteins as indicated in Figure 1. The intracellular level of A3G was efficiently reduced by WT Vif (Figure 1C, lane 2) when compared to the vector control (Figure 1C, lane 1). Except Vif 1-176, other Vif truncated mutants had an impaired ability to reduce the expression of A3G (Figure 1C, lanes 4–8). Accordingly, these Vif truncated mutants had a reduced ability to exclude A3G from virions and to suppress the antiviral activity of A3G when compared with WT Vif (Figure 1C, lanes 4–8 and 1D). These data suggested that Vif truncated mutants lacking the BC box or having a disrupted HCCH region lost the ability to counteract A3G.

### Specific amino acids in HCCH motif of HIV-1 Vif mediate Vif-CBF-β interaction

**Figure 2:**
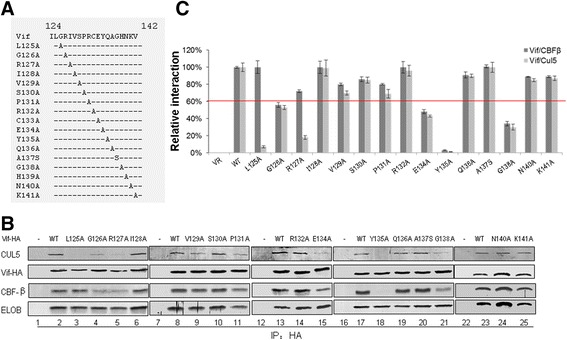
**Certain residues in HCCH region of Vif are important for interaction with CBF-β and/or CUL5. (A)** Illustration of Vif mutant constructs. **(B)**. Amino acids 124-141 located in HCCH region of Vif involved in CBF-β/CUL5 interaction. HEK293T cells were transfected with VR1012 as a control vector or WT or various Vif mutants as indicated. Cells were harvested 48 h later and subjected to immunoprecipitation analysis using the anti-HA antibody conjugated to agarose beads. Co-precipitated proteins were analyzed by Western blotting against Vif-HA, CUL5, CBF-β and ELoB. **(C)** Relative binding capacity of WT Vif (100%) and Vif mutants to CBF-β or CUL5. Error bars represent the standard deviation from triplicate experiments.

As shown above, Vif 1-141 bound to CBF-β with reduced binding affinity when compared to WT Vif, while Vif 1-124 completely lost its interaction with CBF-β, suggesting that certain residues from amino acids 125–141 are required for Vif binding to CBF-β. We thus generated a series of Vif mutant constructs in which individual amino acid residues were replaced with alanine or serine in this region (Figure 2A). HEK293T cells were transfected with VR1012 as a negative control vector or WT or various Vif mutants as indicated in Figure 2B. Interactions of the WT or Vif mutants with endogenous CUL5, ELOB and CBF-β were examined by co-immunoprecipitation. As previously shown [22], CUL5, ELOB and CBF-β were readily co-immunoprecipitated by the HA-tagged WT Vif protein (Figure 2B, lanes 2, 8, 13, 17 and 23).

We found Vif mutants G126A, E134A and G138A were defective at different levels in binding to CBF-β (Figure 2B, lanes 4, 15 and 21), and Y135A completely failed to interact with CBF-β (Figure 2C, lane 18). Accordingly, these mutants exhibited a marked decrease in CUL5 interaction. These results are consistent with previous reports demonstrating that CBF-β regulates the Vif-CUL5 interaction [20-24,48,49]. Unlike with the G126A, E134A, Y135A and G138A mutants, Vif L125A showed an impaired interaction with CUL5 but not CBF-β (Figure 2B lanes 3), while the R127A mutant was defective in binding to both CUL5 and CBF-β, with a stronger effect observed on the Vif-CUL5 interaction than the Vif-CBF-β interaction (Figure 2B lanes 5). Thus, L125 and R127 may mainly regulate CUL5 binding, consistent with the published crystal structure of Vif-CBF-β-ELOB/C-CUL5 [54] showing direct interactions of Vif L125 and R127 with CBF-β. In repeated experiments in the current study, the G126A, E134A, Y135A and G138A mutants showed 40–90% reduction in binding to CBF-β and CUL5 when compared with WT Vif; meanwhile, L125A and R127A showed 80–90% reduction in CUL5 binding and 0–30% reduction in CBF-β binding (Figure 2C). The findings here are consistent with our previous observation that CUL5 silencing did not affect the Vif-CBF-β interaction, while CBF-β silencing affected the binding of Vif to CUL5 [22]. Thus, the identification of the importance of HIV-1 Vif G126, E134, Y135 and G138 in CBF-β binding further confirmed that the region from amino acid 125–141 at the C-terminus of HIV-1 Vif is required for the Vif-CBF-β interaction.

### HCCH motif in HIV-1 Vif affects Vif-CBF-β interaction

**Figure 3:**
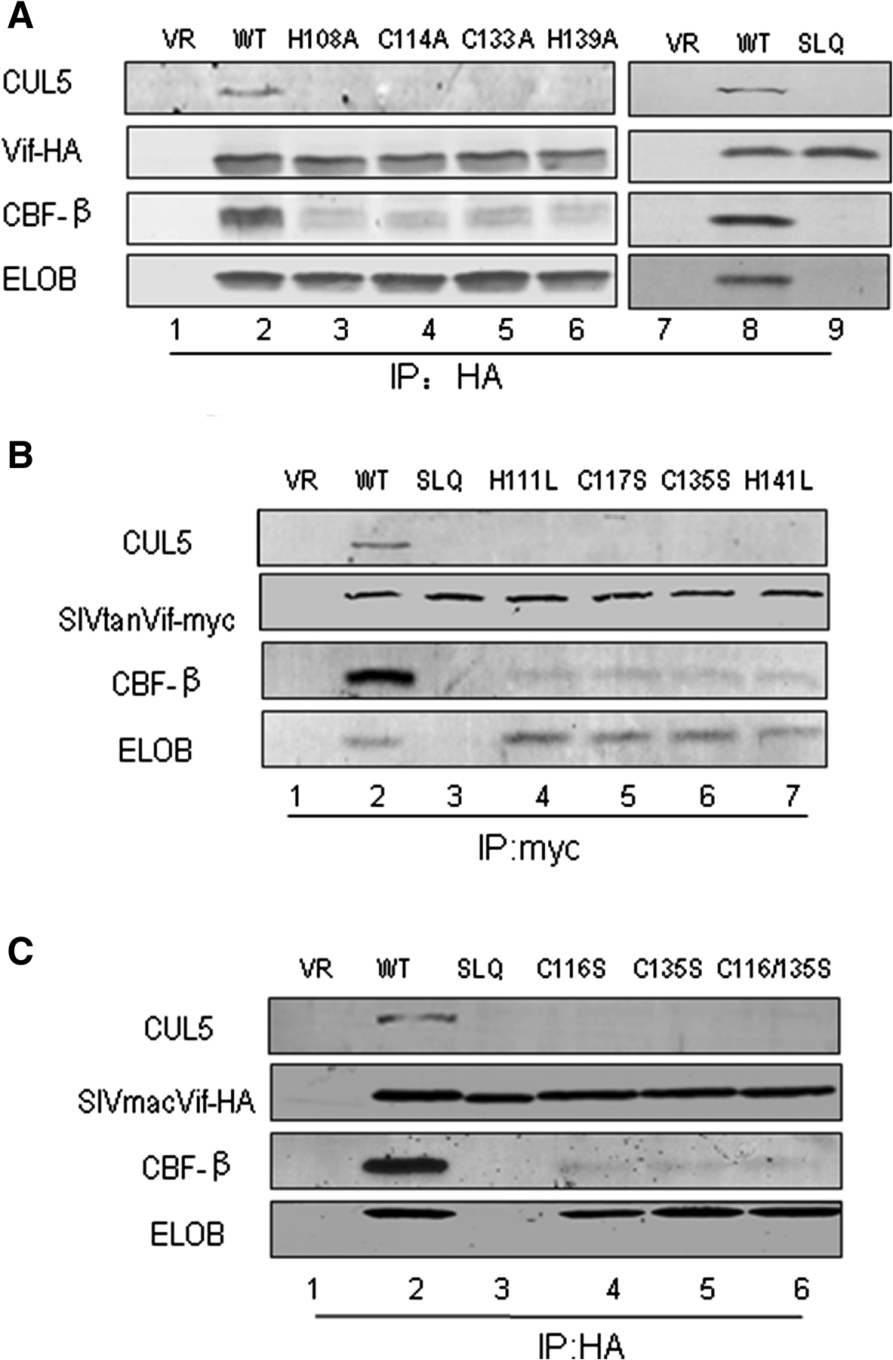
**The HCCH motif in primate lentiviral Vif proteins is involved in CBF-β/CUL5 interactions.** HCCH or SLQ-AAA mutants in HIV-1 Vif **(A)**, SIVtan Vif **(B)** or SIVmac Vif **(C)** were transfected into HEK293T cells as indicated. Cells were harvested 48 h later and subjected to immunoprecipitation analysis using the anti-HA antibody conjugated to agarose beads. Co-precipitated proteins were analyzed by Western blotting against Vif-HA, CUL5, CBF-β and ELOB.

After determining above that certain residues in the HCCH region affected the Vif-CBF-β interaction, we wondered whether the entire HCCH motif which is known to affect the Vif-CUL5 interaction would also influence the Vif-CBF-β interaction. This hypothesis was investigated by transfecting HEK293T cells with VR1012 as a negative control vector or expression vectors for WT Vif or various Vif mutants as indicated in Figure 3A. As expected, the H108A, C114A, C133A and H139A mutants could not interact with CUL5 (Figure 3A, lanes 3, 4, 5 and 6). Moreover, the H108A, C114A, C133A and H139A mutants also showed a decreased interaction with CBF-β, suggesting that these residues, which were previously shown to stabilize the conformation of the Vif α-domain [54], not only affect the interaction with CUL5 but also contribute to Vif binding with CBF-β. We next detected whether the SLQ-AAA mutation, which affects the Vif-ELOB/C interaction [30], has an influence on Vif binding with CBF-β. The results showed that the SLQ-AAA mutant completely lost the ability to interact with CBF-β (Figure 3A, lane 9) when compared with WT Vif (Figure 3A, lane 8). Primate lentivirus Vif proteins have been shown to all require CBF-β to degrade their host restriction factors. In order to determine whether SLQ-AAA and the HCCH motif in SIVtan and SIVmac Vif proteins have a similar effect on the interaction between Vif and CBF-β, we transfected VR1012 as a negative control or expression vectors for WT SIVtan Vif or various SIVtan Vif mutants into HEK293T cells in six-well plates. Similar to the HCCH motif in HIV-1 Vif, the HCCH motif in SIVtan Vif (Figure 3B, lanes 4, 5, 6 and 7) also showed a very weak interaction with CBF-β compared with WT SIVtan Vif (Figure 3B, lane 2). SIVmac Vif also showed similar results to those of HIV-1 and SIVtan Vifs. These results indicated that the HCCH motif in primate lentivirus Vif proteins also contribute to binding with CBF-β.

### Certain residues in HCCH motif of HIV-1 Vif are required for suppression of A3G and A3F

**Figure 4:**
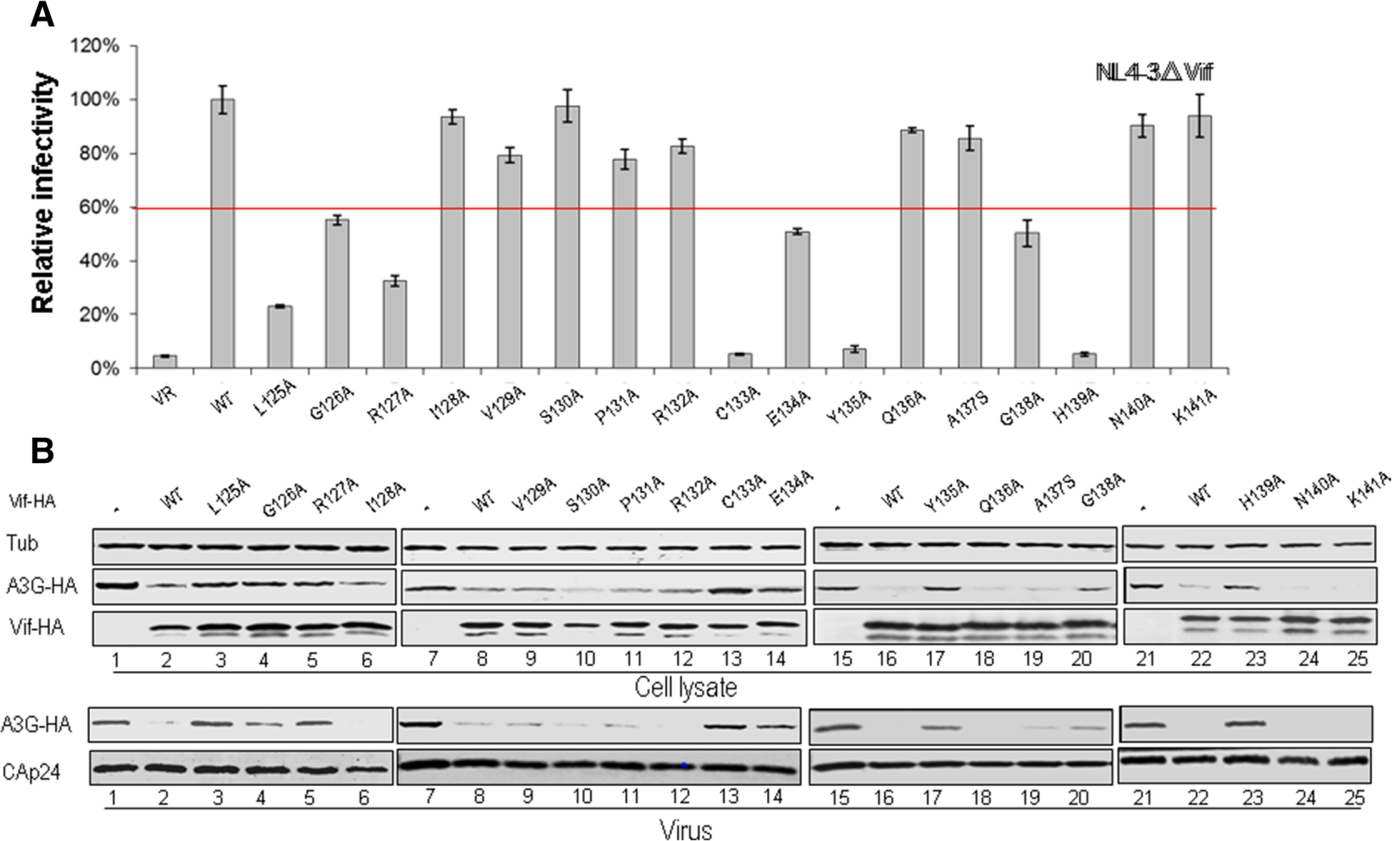
**Effects of Vif mutations in the region from amino acid 124-141 on the antiviral activity of A3G. (A)** Effects of WT and mutant Vif proteins on A3G antiviral activity. HIV-1 viruses were produced by transfecting HEK293T cells with NL4-3ΔVif and A3G along with VR1012 as a control vector or WT or various Vif mutants as indicated. Virus infectivity was assessed using MAGI indicator cells, with the virus infectivity in the presence of WT Vif set to 100%. Error bars represent the standard deviation from triplicate wells. **(B)** Effects of WT and mutant Vif proteins on A3G degradation and virion packaging. A3G expression was assessed by Western blotting against A3G-HA, Vif-HA and tubulin as loading control. A3G packaging was evaluated by Western blotting against A3G-HA and CAp24 after virus was purified from the cell culture supernatant.

After identifying the CBF-β binding residues in the Vif HCCH region, we further analyzed the effect of these Vif mutants on the antiviral activity of A3G proteins. The MAGI assay was used to analyze the function of Vif mutants against A3G (Figure 4). As expected, Vif mutants L125A, G126A, R127A, C133A, E134A, Y135A, G138A and H139A were defective in the suppression of A3G antiviral activity (Figure 4A). As a control, other Vif mutants, including I128A, V129A, S130A, P131A, R132A, Q136A, A137S, N140A and K141A, showed abilities to inhibit A3G comparable to that of WT Vif. To further confirm whether these Vif mutations that abolished the A3G suppression are due to reduced abilities to degrade and exclude A3G from virons, A3G expression levels in cells and virions were analyzed (Figure 4B). The Vif mutants L125A, G126A, R127A, C133A, E134A, Y135A, G138A and H139A (Figure 4B, lanes 3, 4, 5, 13, 14, 17, 20 and 23) were found to be defective in degrading A3G and excluding it from HIV-1 virions compared with WT Vif (Figure 4B, lanes 2, 8, 16 and 22).

**Figure 5:**
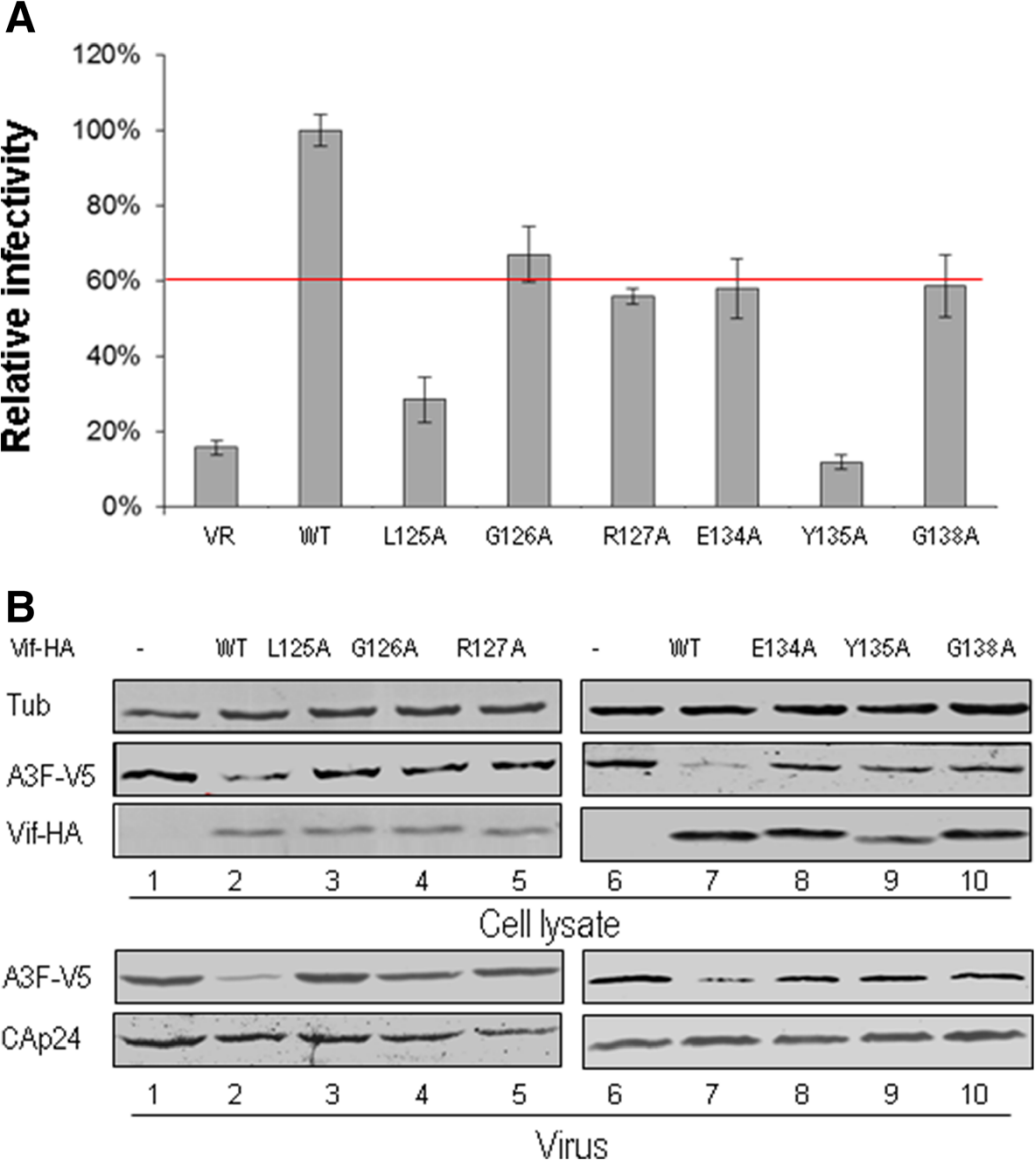
**Effects of Vif mutations in the region from amino acid 124-141 on the antiviral activity of A3F. (A)** Effects of WT and mutant Vif proteins on A3F antiviral activity. HIV-1 viruses were produced by transfecting HEK293T cells with NL4-3ΔVif and A3F along with VR1012 as a control vector or WT or various Vif mutants as indicated. Virus infectivity was assessed using MAGI indicator cells, with the virus infectivity in the presence of WT Vif set to 100%. Error bars represent the standard deviation from triplicate wells. **(B)** Effects of WT and mutant Vif proteins on A3F degradation and virion packaging. A3F expression was assessed by Western blotting against A3F-V5, Vif-HA and tubulin as loading control. A3F packaging was evaluated by Western blotting against A3F-V5 and CAp24 after virus was purified from the cell culture supernatant.

To further confirm whether the identified residues are also important for Vif suppression of another APOBEC3 protein, we also examined the effects of these mutants on the antiviral activity and expression of A3F, with empty vector VR1012 used as a negative control. The Vif mutants L125A, G126A, R127A, E134A, Y135A and G138A were confirmed to have reduced inhibitory effects on A3F antiviral activity relative to that of WT Vif (Figure 5A). The intracellular level of A3F also was restored with these Vif mutants (Figure 5B, lanes 3, 4 5, 8, 9 and 10) when compared to that with WT Vif (Figure 5B, lanes 2 and 7). Additionally, higher levels of A3F were found in HIV-1 virions with Vif mutants (Figure 5B, lanes 3, 4 5, 8, 9 and 10) compared to that with WT Vif (Figure 5B, lanes 2 and 7).

### Mutations in HIV-1 Vif that disrupt the interaction with CBF-β or CUL5 do not influence its interaction with A3G or A3F

**Figure 6:**
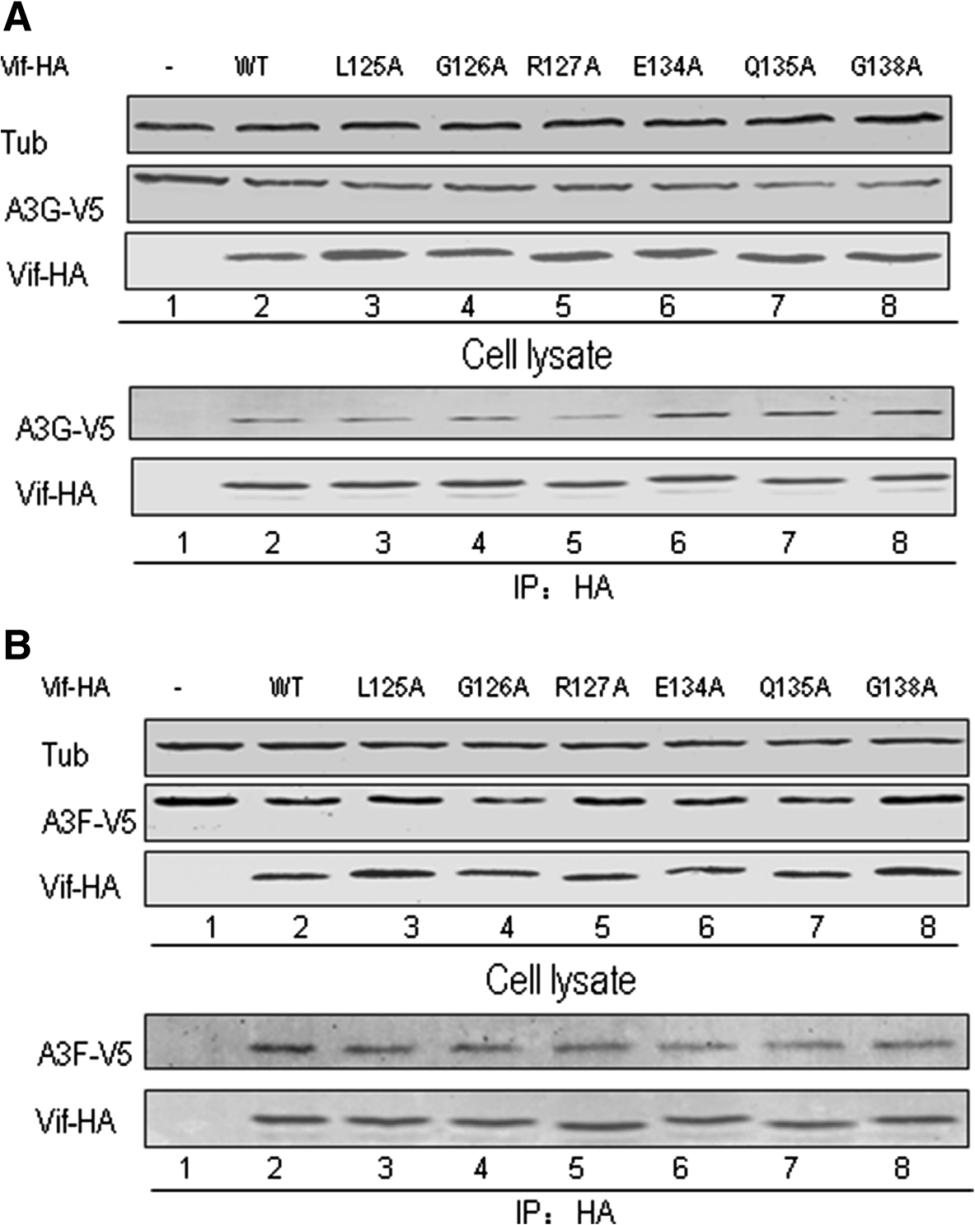
**Unaltered interactions of Vif mutants with A3G or A3F imply no changes in conformation from WT Vif protein HEK293T cells were transfected with the vector control or WT or Vif mutant plus A3G-V5 (A) or A3F-V5 (B).** Cells were treated with 10 μM MG132 12 h prior to harvesting, and then Vif-HA was immunoprecipitated from cell lysates with the anti-HA antibody conjugated to agarose beads. The interaction of Vif-HA with A3G-V5 or A3F-V5 was detected by Western blotting against Vif-HA and A3G-V5 or A3F-V5.

We have shown that some mutations in the HCCH region of HIV-1 Vif affect their function and the Vif-CBF-β or Vif-CUL5 interaction. In order to determine whether these mutations induced conformational changes that abolished the interaction of Vif with CBF-β or CUL5, we further examined the ability of Vif mutants to bind target protein A3G or A3F. HEK293T cells were transfected with negative control vector VR1012 or WT or different Vif mutants plus A3G-V5 or A3F-V5. Cells were treated with 10 μM MG132 12 h prior to harvesting. At 48 h after transfection, cells were harvested, lysed and then loaded onto HA agarose-conjugated beads for immunoprecipitation. All Vif mutants were efficiently co-immunoprecipitated with A3G or A3F similar to results with WT Vif (Figure 6A and B, lanes 2–8). These interactions were specific, since A3G-V5 or A3F-V5 was not detected in the absence of Vif (Figure 6A and B, lane 1). These results suggested that these Vif mutants were not misfolded and the altered amino acids resulted in loss of their interaction with CBF-β or CUL5.

## Discussion

In the current study, we determined that certain residues in the C-terminus of HIV-1 Vif play an important role for binding with CBF-β by detecting the interaction between C-terminal truncated Vif mutants of various lengths with CBF-β. The truncated Vif mutants tested showed binding affinities at different degrees for CBF-β. Compared to WT, Vif 1-176 and Vif 1-150 maintained the WT level of binding with CBF-β, suggesting that the C-terminal region (amino acids 151–192) of Vif is not essential for this interaction. However, Vif 1-141 had a slightly reduced interaction with CBF-β, meanwhile, Vif 1-124 poorly bound to CBF-β, and Vif 1-109 completely lost the ability to interact with CBF-β (Figure 1B). Thus, some residues between positions 125 and 141 located in the HCCH region of Vif, which have been thought to be critical for CUL5 binding [36,54], are also involved in CBF-β binding. Interestingly, our findings are consistent with a previous study which reported that Vif 1-140 retains the WT binding capacity to CBF-β *in vitro* [23]. However, our results are different from those of another study proposing that Vif 1-109 still retains the ability to bind CBF-β but with a reduced affinity by using a co-expression and co-purification system in *Escherichia coli* [50]. As over-expression of proteins *in vitro* increases their chance of interacting with each other, we speculate that a small fraction of Vif 1-109 and CBF-β survived the purification due to the great abundance of expressed proteins.

Notably, the Vif 1-141 truncated mutant missing the BC box still bound to CBF-β, although with minimally reduced binding affinity *in vivo* (Figure 1B). By contrast, the SLQ-AAA mutant totally lost the ability to interact with CBF-β, similar to the result obtained when silencing ELOB (Figure 3) [55]. Others have speculated that Vif binds ELOB/C at its C-terminus followed by CBF-β at its N-terminus, inducing structural changes at both termini. Once Vif is bound to both CBF-β and ELOB/C, CUL5 binds to Vif, requiring residues in both the N- and C-terminus of the protein to assemble a functional ubiquitin ligase [56]. Therefore, the processes of Vif binding to ELOB/C and to CBF-β are closely associated. We deduced that the truncation of the Vif 1-141 mutant may have exposed the binding sites for CBF-β or facilitated the CBF-β interaction. This observation is also consistent with a previous determination that CBF-β could increase the solubility of the Vif 1-140 mutant even without co-expression with ELOB/C [23].

We further identified several residues in the HCCH region of HIV-1 Vif that when mutated affected Vif-mediated A3G and A3F degradation/suppression by disrupting its interaction with CBF-β. Alanine or serine substitution mutants showed that amino acids G126, E134, Y135 and G138 in the Vif 124-141 region (Figure 2) clearly reduced/abolished the CBF-β interaction. Although these residues do not come into contact with CBF-β directly based on the crystal structure [54], they may be required for the conformation of Vif to facilitate CBF-β binding. Mutations of these residues impaired the ability of Vif to suppress A3G/A3F (Figures 4 and 5). However, these Vif mutants did not lose their interaction with ELOB (Figure 2), A3G or A3F (Figure 6), suggesting their conformational changes at least had no effect on the interaction with ELOB and the substrate. Thus, besides an extensive region of the Vif N-terminus, including dispersed and conserved hydrophobic amino acids that were shown previously to bind to CBF-β [22,24,50-52], the finding of C-terminal residues in Vif that also are involved in CBF-β binding further clarified the interaction pattern of Vif-CBF-β. As both the N-terminal and C-terminal domains of Vif function in CBF-β binding, the simultaneous use of additional domains or a specific conformational shape to bind CBF-β and facilitate assembly of the Vif-CUL5 E3 ligase would seem to be required.

Previous studies have established that the HIV-1 Vif HCCH motif is required for binding with CUL5 [33-39]. The zinc-finger motif may stabilize the conformation of the α–domain to promote Vif interaction with CUL5 [50,54]. Mutations of two equivalent HIV-1 Vif residues in HXB2 Vif, I120S and L124S [35], or other key residues have been shown to impair Vif interaction with CUL5. Finding that the HCCH domain may also contribute the interaction of Vif with CBF-β was surprising (Figure 3). Thus, we speculate that the conformational shape of Vif is also required for the CBF-β interaction, which then facilitates CUL5 binding and the assembly of Vif-CUL5 E3 ligase. Essentially, the HCCH domain may have dual roles in CBF-β binding and CUL5 binding.

Until now, we have found that most of the residues that affect the interaction with CUL5 also affect the interaction with CBF-β. Therefore, a specific conformation of Vif may be important for its interactions with CUL5 and even more importantly with CBF-β, which is consistent with data showing that silencing of CBF-β inhibits the interaction between Vif and CUL5 [22]. However, unlike most of the CUL5 binding sites identified previously, here we observed that Vif L125A only affected CUL5 binding but maintained the WT levels of binding to ELOB and CBF-β.

As a substrate receptor, Vif not only recruits ELOB/C, RBX and CBF-β to form an E3 ubiquitin ligase, it also needs to recognize different APOBEC3 proteins for degradation. Different functional domains of Vif have been well characterized. Vif proteins of different HIV-1 subtypes and SIV have all been determined to require CBF-β to degrade A3G [22,48], and the current study further delineates the role of various Vif regions. Identification of G126, E134, Y135 and G138 of HIV-1 Vif in the Vif-CBF-β interaction better defines the Vif functional domain, which may be help to facilitate the development of novel therapeutic strategies for HIV-1 infection.

## Conclusions

Certain amino acids (G126A, E134A, Y135A and G138A) in the C-terminus of HIV-1 Vif were identified as binding sites for the interaction with CBF-β. The HCCH motif itself, which is required for CUL5 interaction, also affects Vif-CBF-β interaction. Furthermore, we speculate that binding to ELOB/C induces conformational changes in Vif, facilitating its interaction with CBF-β and consequent interaction with CUL5. The results provide important information on the assembly of the Vif-CUL5-E3 ubiquitin ligase. Identification of the new binding interface with CBF-β at the C-terminus of HIV-1 Vif also provides novel targets for the development of HIV-1 inhibitors.
